# Project20: interpreter services for pregnant women with social risk factors in England: what works, for whom, in what circumstances, and how?

**DOI:** 10.1186/s12939-021-01570-8

**Published:** 2021-10-24

**Authors:** Hannah Rayment-Jones, James Harris, Angela Harden, Sergio A. Silverio, Cristina Fernandez Turienzo, Jane Sandall

**Affiliations:** 1 Department of Women and Children’s Health, Faculty of Life Sciences & Medicine, King’s College London, 10th Floor, North Wing St. Thomas’ Hospital, Westminster Bridge Road, London, SE1 7EH UK; 2 Chelsea and Westminster NHS Foundation Trust, Clinical Research Facility, St Stephen’s Centre, 252 Fulham Road, London, SW109NA UK; 3 School of Health Sciences, City, University of London, Northampton Square, London, EC1V 0HB UK

**Keywords:** Maternity services, Interpretation, Translation, Non-English speaking, Language barriers

## Abstract

**Background:**

Black and minority ethnic women and those with social risk factors such as deprivation, refugee and asylum seeker status, homelessness, mental health issues and domestic violence are at a disproportionate risk of poor birth outcomes. Language barriers further exacerbate this risk, with women struggling to access, engage with maternity services and communicate concerns to healthcare professionals. To address the language barrier, many UK maternity services offer telephone interpreter services. This study explores whether or not women with social risk factors find these interpreter services acceptable, accessible and safe, and to suggest solutions to address challenges.

**Methods:**

Realist methodology was used to refine previously constructed programme theories about how women with language barriers access and experience interpreter services during their maternity care. Twenty-one longitudinal interviews were undertaken during pregnancy and the postnatal period with eight non-English speaking women and their family members. Interviews were analysed using thematic framework analysis to confirm, refute or refine the programme theories and identify specific contexts, mechanisms and outcomes relating to interpreter services.

**Results:**

Women with language barriers described difficulties accessing maternity services, a lack of choice of interpreter, suspicion around the level of confidentiality interpreter services provide, and questioned how well professional interpreters were able to interpret what they were trying to relay to the healthcare professional during appointments. This resulted in many women preferring to use a known and trusted family member or friend to interpret for them where possible. Their insights provide detailed insight into how poor-quality interpreter services impact on their ability to disclose risk factors and communicate concerns effectively with their healthcare providers. A refined programme theory puts forward mechanisms to improve their experiences and safety such as regulated, high-quality interpreter services throughout their maternity care, in which women have choice, trust and confidence.

**Conclusions:**

The findings of this study contribute to concerns highlighted in previous literature around interpreter services in the wider healthcare arena, particularly around the lack of regulation and access to high-quality interpretation. This is thought to have a significant effect on pregnant women who are living socially complex lives as they are not able to communicate their concerns and access support. This not only impacts on their safety and pregnancy outcomes, but also their wider holistic needs. The refined program theory developed in this study offers insights into the mechanisms of equitable access to appropriate interpreter services for pregnant women with language barriers.

**Supplementary Information:**

The online version contains supplementary material available at 10.1186/s12939-021-01570-8.

## Introduction

Although the majority of the foreign-born UK population reported speaking English well or very well in the most recent census [1], around 9% of the population in areas with high diversity such as parts of London, Leicester and Birmingham reported not being able to speak English well or at all. In fact research has suggested that London is one of the most linguistically diverse cities in the world with over 300 reported languages [2] with the number of non-English speaking population increasing substantially since the last census [3]. There is a wealth of evidence associating language barriers with disadvantage and inequality including increased poverty, employment, multiple health issues and adverse events when accessing healthcare services [1, 4–6]. Pregnant women who do not speak fluent English are at greater risk of poor birth outcomes compared to their English-speaking counterparts and intersecting factors such as racial discrimination, poverty, housing issues, poor mental health further exacerbate this risk [1, 7–10]. When women with these complex social risk factors are unable to effectively communicate with their healthcare provider, many of their needs remain unknown and unmet [11].

In the wider healthcare arena barriers to effective communication with healthcare professionals have been identified despite increased access to interpretation services. Previous research has highlighted that patients with language barriers who access general healthcare services are less likely to actively participate in their care, do not share their concerns, ask fewer questions and are less verbally dominant than patients belonging to the majority population [8, 12, 13]. A study carried out across a number of UK general practices found that professional interpreter services including face-to-face interpretation and telephone interpretation were under-utilised, with bilingual healthcare professionals or family and friends being used for most consultations [14]. This type of informal interpretation can be problematic if the language skills are poor, when sensitive issues are being discussed, or when disclosure of safety issues such as domestic violence can put the service user at risk. Although not a panacea for language barriers, professional interpreter services have been found to improve effective communication and clinical care [15]. Less is known about how pregnant women with language barriers experience maternity services and the interpretation they offer, but inequalities in their outcomes suggest similar issues around their ability to seek help and access safe and appropriate care [16]. An Australian study of pregnant immigrant women found that they were more likely than Australian born women to be listened to and receive adequate information during pregnancy [10]. A realist synthesis of how women with social risk factors experience their maternity care in the UK found that their needs for interpreter services were sometimes overlooked by healthcare professionals resulting in compromised safety and inequity of information received. A programme theory was put forward that suggested a need to improve access to interpretation services throughout the antenatal, intrapartum, and postnatal period, including emergency admissions. The National Institute of Clinical Excellence [17] called for research into pregnant women’s perceptions and experiences of interpreter services to be able to design effective interventions that increase their participation and in turn, safety. Therefore study aimed to explore how non-English speaking women with social risk factors experience their maternity care, and whether or not they find interpreter services offered acceptable, accessible and safe. The findings will enable the refinement of a programme theory to provide practical insights that improve their ability to communicate concerns and sensitive issues through the use of professional interpretation services.

## Methods

### Realist methodology

Realist methodology is a theoretically informed, pragmatic approach to evaluating an intervention or programme that often uses mixed methods to understand how the intervention is working or not working in different contexts [18]. A realist question is not ‘does it work?’ but ‘how, for whom, in what circumstances does it work’? This allows those implementing interventions to refine, scale-up, or even withdraw the service [19]. Realist methodology is typically used in the evaluation of complex interventions, which is why it is particularly suited to exploring the mechanisms of interpreter services for women with complex needs, within a complex health system. The pragmatic nature of the realist approach attempts to cut through this complexity to focus on the most important aspects of the intervention that usually focus on the human response [20]. Theories about how an intervention is thought to be working are tested, refined, and articulated through context (C) + mechanism (M) = outcome (O) configurations (referred to as ‘CMO’ configurations) to provide specific, practical recommendations [19].

The aims of this research were approached through the testing and refinement of a programme theory (PT) constructed from a realist synthesis of how women with social risk factors experience UK maternity care, focus groups with midwives and service user engagement. Although part of a wider evaluation of specialist models of maternity care (https://www.project20.uk), the PT tested in this study relate to women’s access to interpreter services during their maternity care, and their experiences of those services offered see Table 1:

**Table 1:** Initial programme theory

**Context**	**Mechanisms**	**Outcomes**
Women who do not speak English and those who have difficulties communicating (learning or physical disabilities).	**1)** Uncomplicated telephone access to interpreter services, or online provision to register with services, arrange or reschedule appointments, organise travel to appointments and to access advice from a healthcare professional. **2)** Access to properly translated, language appropriate materials. **3)** Choice of interpreter, for example a female, an anonymous, or a trusted interpreter. **4)** Access to interpretation services throughout antenatal, intrapartum and postnatal period, including emergency admissions.	Earlier access to services, avoidance of denial of service, improved safety, flexibility, equity in information received, increased confidence in help seeking and self-disclosure.

The programme theory was tested through analysis of 21 semi-structured, longitudinal interviews, with eight non-English speaking women with social risk factors, and their families. All women interviewed were under the care of one of two specialist models of maternity care that involved continuity of carer. One specialist model (CBM) took a community-based approach and was placed within an area of significant health inequality. The other model (HBM) was based within a hospital setting and provided care for women based on an inclusion criteria of social risk factors. The initial programme theory relating to interpreter services were incorporated into a realist informed interview guide and refined using thematic analysis that identified specific contexts, mechanisms and outcomes. The refined programme theory will be useful to those developing maternity services and interpreter services for women with language barriers.

### Setting

Two inner city National Health Service (NHS) maternity service providers in the UK that provide care to a multi-cultural, socioeconomically diverse population were purposively selected. Each provider offered a telephone-based interpretation service.

### Data collection

Semi-structured, longitudinal interviews were carried out in a setting of the woman’s choice at around 28- and 36-weeks’ gestation, and 6-weeks after birth. The women’s family members and friends were also invited to participate in the interviews to give additional insight. Through purposive sampling, women were identified by the specialist model midwives providing their care if they met the following inclusion criteria:

- Low socio-economic status (SES) calculated by an Indices of Multiple Deprivation [IMD] score [21] of higher than 30 AND/OR secondary school as the highest level of education attained.

The IMD score was calculated using the woman’s postcode to give a composite measure using routine data from seven domains of deprivation [22] to identify the most disadvantaged areas in England. Level of education was self-reported and categorised into three groups: no completed education or completed only primary school; completed secondary school; and completed tertiary (university or college). The highest level of education attained was chosen as an indicator of deprivation as it has a clear influence on occupational opportunities and earning potential [23]. Indicators measuring life course socioeconomic position, for example income, housing, relationship and occupation, and any social risk factors were also collected and reported. Social risk factors were not included in the criteria as the research aimed to explore whether or not women are more likely to disclose social risk factors during their pregnancy if they received care from the specialist model. That said, all women were experiencing at least one social risk factor in addition to low SES and/or limited education.

Interviews were undertaken by a realist-interview trained academic and midwife using Manzano’s [24] approach to refine programme theories and improve rigour through the ‘teacher-learner’ relationship. In this case the interviewer presented theories extracted from a realist synthesis [11] and asked the women about their experiences to confirm, falsify, explain and refine the theories. See supplementary file 1 for the full interview guide and programme theories tested. The women’s insights are not considered to be constructions, but ‘evidence for real phenomena and processes’ [25] that contribute to the overall evaluation of the programme’s effectiveness. The realist-informed interview guide included in supplementary file 1, allowed for both the testing of pre-constructed theories, and new programme theories to emerge.

During the qualitative interviews, a range of interpretation methods were used depending on the participants choice including professional telephone interpreters, family members, and other healthcare professionals or researchers who were able to speak the woman’s native language. These different methods over the course of the longitudinal interview schedule provided an opportunity for women to discuss their experience of different methods of interpretation openly as trust developed. The realist trained interviewer was present at all interviews and those conducted by a native language speaker were interpreted in English for transcription purposes. Verbatim transcription of interview data were carried out by an external source.

### Data analysis

For the purpose of this paper, only data relating to language barriers and interpreter services were analysed. The qualitative data were coded using NVivo v.12 and analysed using a thematic framework analysis [26]. This method, and software, allowed for the organisation of a large qualitative dataset into a coding framework matrix, developed using the previously constructed programme theories [11, 27], and to uncover new theories. It also allowed us to see the differences in women’s experiences depending on their individual contexts [26]. Validity was strengthened through the use of patient and public involvement group who assessed interview transcripts and highlighted where the data confirmed or refuted the initial programme theories, as well as the emergence of new theory.

Women receiving the community-based specialist model of care are identified using ‘CBM’ followed by a number, and those receiving the hospital-based specialist model ‘HBM’, allowing for the analysis of differences between place-based care. Two members of the research team read and re-read each transcript thoroughly and assigned sections of the text to the programme theories. Similar codes were grouped under higher-order categories to unearth middle range theories such as access and choice of interpreter. We utilised existing models of data adequacy [28] to assess acceptable data quality.

## Results

### Participants

Eight non-English speaking pregnant women with low socio-economic status and/or educational attainment and at least one social risk factor were recruited along with three friend or family members- See Table 2. All women were under the care of a specialist maternity model that aimed to provide antenatal, intrapartum and postnatal continuity of care. Only three participants were first time mothers, but for four of the five multiparous women, this was their first pregnancy in the UK. Based on the 2019 IMD scores [22], all participants lived in the 1st or 2nd most deprived deciles. All participants were experiencing between one and seven social risk factors including mental health issues, domestic violence, single motherhood, financial and housing problems, previous sexual abuse/trafficking, female genital mutilation and no recourse to public funds. Five participants were seeking asylum, had refugee status, or had had an asylum claim refused. In addition to these risk factors some participants had experienced other highly traumatic events including fleeing from a war-torn country, the kidnap of a close family member, had been held in an immigration detention centre, dispersal, had children removed from their care, and childhood sexual abuse.

**Table 2:** Characteristics of women interviewed

**Characteristic**	**Community based model (CBM)** ***n* = 4**	**Hospital based model (HBM)** ***n* = 4**	**Total** ***n* = 8**
**Ethnicity and migration status**			
Asian	0	2	2
Black African	3	0	3
Black Caribbean	0	0	0
White Other	1	2	3
Asylum seeker/refugee	2	3	5
**Age**			
18–24	0	1	1
25–29	0	0	0
30–34	2	2	4
> 34	2	1	3
**Parity**			
Primiparous	2	1	3
**IMD Decile (2019)**			
Most deprived 1st +2nd	4	4	8
**No of social risk factors (excluding language barrier)**			
1	0	0	0
2	1	0	1
3	1	1	2
4	1	0	1
≥ 5	1	3	4

### Findings

Analysis of the women’s interview data provided detailed insight into barriers to access and how poor-quality interpreter services impact on women’s ability to seek help, disclose risk factors and communicate effectively with their healthcare providers. Firstly, programme theories and rival theories relating to women’s access to, choice and experience of interpreter services are presented, followed by relevant quotations from the qualitative data, concluding with the refined programme theory:

#### Initial programme theory

If women have easy, immediate telephone access to interpreter services to register with maternity services, arrange or reschedule appointments, organise travel to appointments, and access to properly translated materials, then inequity in information received and a key communication barrier will be overcome, and women will be better able to access and engage with services.

#### Initial programme theory

If HCP’s listen to women’s choices about interpreter services, for example a female, an anonymous, or a trusted interpreter, then barriers to their use and effectiveness will be reduced and women would feel more comfortable discussing sensitive subjects and disclosing concerns with their healthcare provider, improving safety.

#### Rival theory

If women do not trust discussing personal matters with an interpreter, despite whether the interpreter was a stranger or someone from within their own social community, then language barriers will continue, and women will not disclose sensitive information.

#### Testing using qualitative data

##### Access to interpreter and maternity services

The ability to contact services to book or rearrange appointments or seek help over the phone was identified as a problem for some women.

*I think contacting the GP it’s more difficult usually because of the language barrier… it’s truly my weakness here and it’s easy if I don’t have to call, and I can be just given the appointments. I’m much more comfortable that way. I haven’t had any need to contact the midwife yet so far. If I have to contact her in the future I may just need to use a friend to help me to contact her (CBM5)*

*‘Not [offered interpreter services] within my pregnancies… I used it in the NHS for… how you say? Psychiatric?... Yeah it was helpful. Yeah because I have to … tell many things that I don’t normally talk to anyone… and that was very hard for me so it’s, it’s hard anyway for me to translate, like properly, you know?’ (HBM10)*

Other women described the benefits of having a healthcare professional that could speak their native language or antenatal classes provided in their native language:

*‘No they don’t offer it [interpreter service] but if I really need it, yeah I will ask for them… The good thing about this is that [specialist model midwife] speaks Spanish…. that was very helpful that she can explain, you know, because she speaks the same language as me.’ (HBM10)*

*‘I attended antenatal classes, um, one day so [name of hospital] provide, um, antenatal classes in Spanish, so that was very useful, um, with a lot of people who speak Spanish from, you know, Latin America, Spain, and it was, it was beautiful, it was interesting, and um, but I do feel it was a bit short, it was just one day so I felt, you know, it would be very very useful to have at least another day at least a couple of days, and um, yeah it was an opportunity, you know, to share experience and opinions and love.’ (CBM5)*

A woman’s family member described the difficulties non-English speaking people face in registering with health services.

*‘I’m a builder so I have like million friend, I ask them: they don’t all speak English, they won’t use the phone… there’s some of my friends they don’t have a GP. They are here for five years. They don’t know how to open a GP. They don’t know how to fill a form in.’ (HBM6 Family member)*

##### Choice of interpreter

Most of the women interviewed preferred a family or friend to interpret for them as they could trust them. When this was not possible they discussed not having a choice in the interpreter they get, for example a female interpreter when discussing intimate details, or a or face-to-face service.

*‘Most of the time I’m happy, my husband has been able to interpret for me. [*When using professional interpreter services*] I prefer to speak with… women rather than men but they didn’t give me an option they would say, ‘OK, we have this interpreter,’ that’s it and they will call anybody, they didn’t give me any other options. For me I don’t like it, I only like it if it my husband or my close family. (CBM2)*

*‘It’s not always possible and people is working but yes I do rather prefer to have a family member or a friend. Perhaps it will be useful to have physical interpreters, just the person being there with you, um, rather than online, telephone interpreters. I think it’s more useful, you have the person just next to you, you can see, you can talk, and it just inspires more security and trust, than the telephone line.’ (CBM5)*

One woman and her partner gave insight into the potential barrier of family members discouraging the use of interpreter services.

*‘She feels she needed but I, I always tell her that, ‘Don’t use them, because she understand everything… her English is better than mine, but she studied English… She’s shy to speak, she’s shy to communicate. So I always tell her that, ‘Don’t use the interpreter, you don’t need.’ (HBM5 Family member)*

This was confirmed by the woman who described wanting to use interpreter services to be able to properly express herself, but not continuing to do so at her partners wish:

*‘I have tried, er, once, but my husband told me if you get an interpreter you will never speak English... He always told me to , ‘Try, try, try to speak English. Because if you, do it, if you get an interpreter you will depend on her. So don’t do that. Speak English.’ I’ve told him [laughs] many times, sometimes I can’t express myself… There is so much… he [partner who recently attempted suicide and is on antidepressants] had so much problems in taking the medicines, I’ve tried to, to, to tell them [midwives] but, you know, my English is not that much… so I have this difficulty’ (HBM5)*

##### Experience of using interpreter services

Many women questioned how well professional telephone interpreters were able to interpret what they were trying to relay to healthcare professional during appointments:

*I can’t say that all interpreters say what you are really saying. I think about 60% of them are quite accurate and they are explicit in what you are saying, but about 40% of them are more… short, they are not really translating what you are saying… they just change your own words. (CBM5)*

*From my point of view they don’t have the right, whatever I say to you, as the interpreter you have to directly translate the language, you don’t change. But they were saying, ‘No, you can’t say it like this. We need to say it like that.’ … it generally works well, the interpreter services, er, sometimes a proportion of them are a bit direct and I feel they are not translating exactly what I’m saying. Um, at least for the, um, little English that I can speak. Um, and sometimes they are much more direct… they don’t, yeah they don’t translate exactly what I’m saying. (CBM2)*

This appeared to be the case for women from some countries, particularly Black African women, highlighting that interpretation services do not guarantee a level playing field. Some languages are regularly disadvantaged:

*Because the interpreter sometimes they don’t know what you said, they don’t speak … as you said. The interpreter didn’t say exactly what I did and I that’s why I want to do my things myself. Because sometimes they make a mistake. Because of my French, there is French of Ivory Coast, French of the Congo, they speak it different. (CBM7)*

*Sometimes the interpreters don’t really tell what you really feel, the way you tell it. It’s so different. It can be so abstract. I tried using them in, not in my appointments, but doing my paperwork with the government and I had to stop him, and I tried to do it all myself because it’s only me who can, you know, reach the words properly about how I feel and how things was. They can change just one Arabic word and the whole sentence is so different (HBM6)*

Poor experiences and questionable quality of interpretation impacted on women’s reluctance to use interpreter services:

*From my point of view I’m not happy, sometimes you know the interpreter they don’t know what you say, you can see the difference… you can feel it because when you hear them, they didn’t say what you say to then,. I’m not saying all, some of them they are really acting professional, they know what they are doing, some they don’t know. They will say what you didn’t say to them. Because [that’s what] I have experienced, so that’s why personally I don’t like it, I stop it… it’s not fair you see getting money, if he [interpreter] doesn’t know the language, it’s better to say, ‘OK I can’t deal with that one.’ Because in order to get money, don’t put somebody’s life at risk. (CBM4)*

One woman described an experience when an interpreter did not listen to her, resulting in her not being told information at an ultrasound scan appointment:

*There was an interpreter at the scan, but, um, it was very weird because it was a male interpreter and I don’t know if he was really attentive, um sensitive. During the appointment he was talking a lot to the sonographer rather than with me. So I didn’t know if it was a boy or girl. I did ask because I believe I could hear the sonographer saying something about the sex… but he just ignored me, so I still don’t know. (CBM5)*

Confidentiality was also identified as an issue, with women being suspicious about how confidential professional interpreter services are and concern about the opinions of the interpreter.

*‘Is it really confidential? And then if they can resolve your problem when you speak to them as well sometimes, they will say confidential but if it’s not confidential I don’t feel comfortable to speak in front of the interpreter. Because I have a bad experience of the interpreter from Africa… I wasn’t happy about what they were thinking.’ (CBM2)*

The findings presented confirm aspects of the initial programme theories and the rival theory relating to women being given a choice of who interprets for them at appointments and how much trust they have in the service. This should be considered in line with guidance around women being given an opportunity to disclose personal matters away from family members. These insights are incorporated into the refined programme theory- See Table 3.

**Table 3:** Refined programme theory

**Context**	**Mechanism**	**Outcome**
Women who do not speak English and those who have difficulties communicating (learning or physical disabilities). These women are often unfamiliar with the UK health system.	M1) If women who don’t speak English have access to language appropriate information about how to access a GP and maternity services, and help is given to fill in registration forms	O1) Then access to maternity care will not be denied or delayed through the process of registering for NHS services, and women will feel more supported from the beginning of their care experience.
	M2) If women have access to high quality interpreter services during antenatal, intrapartum and postnatal care, education, and are able to book or rearrange appointments and seek help when concerned using translation technology	O2) Then inequity in information received and a key communication barrier will be overcome, women will feel better supported and listened to, be better able to access services and seek appropriate help in a timely manner.
	M3) If women are able to request a different interpreter or method of interpretation if they do not feel the interpretation is accurate or confidential	O3) Then the quality of the interpretation can be improved, and women will have more confidence and trust in the persons providing the service, leading to more meaningful communication with the healthcare professional.
	M4) If healthcare professionals listen to women’s choices about interpreter services and offer preferred options, for example a female, an anonymous, or a trusted interpreter such as a family member or friend	O4) Then barriers to the use and effectiveness of interpreters will be reduced and women would feel more comfortable discussing sensitive subjects and disclosing concerns with their healthcare provider, improving safety.

## Discussion

This study highlights important issues with interpretation services commonly used across the National Health Service that have enabled the refinement of a programme theory to improve women’s access and experience. Initial programme theories relating to interpretation services were constructed from a realist synthesis [11], focus groups with midwives [27], and service user engagement. Testing of these theories through longitudinal interviews with women throughout their pregnancy and postnatal period provided greater insight and depth to the issues women face with accessing maternity services. Overall women described a negative experience of interpreter services during their maternity care, either through a lack of access to the service, or poor-quality interpreter services. This is despite receiving a specialist model of maternity care that incorporated continuity throughout the antenatal, intrapartum and postnatal period. This is an important insight as it should not be assumed that women with language barriers are protected by specialist models of care alone. There did not appear to be a difference in the experiences of interpreter services for women accessing care in the community or hospital setting.

Although guidance states women should be routinely offered interpretation services during antenatal appointments [17], women in this study described difficulties accessing maternity services to arrange appointments or seek help due to their language barrier. As found in previous research [11, 13] this was often due to not being offered interpreter services when healthcare professionals assumed a sufficient level of English. This was a particular issue when trying to book and rearrange appointments, seek help over the telephone, during labour care, or emergency admissions. This highlights potential mechanisms that lead to their poorer engagement with services [29, 30] and inequalities in clinical outcomes [31, 32]. Women also described a lack of choice of interpreter, suspicion around the level of confidentiality interpreter services provide, and most worryingly questioned how well ‘professional’ interpreters were able to interpret what they were trying to relay to the healthcare professional. This resulted in many women preferring to use a known and trusted family member and friend to interpret for them where possible. Another potential issue highlighted was when family members discouraged the use of interpreter services despite women feeling they would benefit from being able to properly articulate themselves. Although evidence around how women experience interpreter services during pregnancy is sparse the use of family members works against the advice that many healthcare professionals try to adhere to [33]. It is recommended that women are seen at least once on their own during pregnancy to give them an opportunity to disclose sensitive issues that they may not be able to in front of family members, for example information about previous pregnancies, terminations, sexually transmitted diseases, drug and alcohol consumption, domestic abuse and physical and mental health issues [34–36]. If women do not trust the interpreter service used during this protected time then it is speculated that they are unlikely to feel able to disclose these highly sensitive issues. This has significant safety implications for women who are already known to be at risk of poor clinical and social outcomes.

Given the insights of the women interviewed in this study, it is suggested that all women are made aware of the possibility to self-refer directly to maternity services by administrative staff at the first point of contact with health services, using language appropriate information, interpretation and translation technologies. Although it is not currently recommended for use in maternity practice and should not be trusted for important medical communication, translation technology such as Google translate© has been evaluated and found to be a useful initial communication tool in healthcare services [37, 38]. Future research should assess the practicability, acceptability and safety of this technology for women’s access to maternity care. An evaluation of the quality of telephone interpreter services is also recommended to address the safety concerns raised in this study and the wider interpreter service research [13]. Until this work is carried out women should be able to report suspected poor translation and have a choice in the interpreter used during their maternity care. Mechanisms associated with improved experiences also included antenatal classes in different languages. Although this is not a realistic option for all languages and contexts it could be achieved in multicultural settings with antenatal support groups or classes in languages common to the local area.

## Limitations

As clearly evident in the findings around women’s experiences of interpreter services discussed previously in this chapter, using the telephone translation service ‘Language Line’ to conduct qualitative interviews with women and their family members may have impacted on the richness and rigour of the interview. This appears to have been overcome for some women when bilingual healthcare professionals or members of the research team were able to conduct the interviews ‘face-to-face’. The limitations of telephone translation services including the potential lack of quality and participants suspicion of the nature of confidentiality when using the service should be considered in future research. All women were given a choice of interpretation and some women chose a family member to interpret for them, as discussed above, this can present limitations in women’s ability to discuss sensitive issues. However, during the course of the longitudinal interview process all women used at least two forms of interpretation, lessening the potential effect of these limitations. The study was limited to the insight of 8 women and their family members, all of whom were experiencing a specialist model of maternity care that may have impacted how they experience interpreter services compared to women accessing standard UK maternity care. A larger study that involves the insights of healthcare professionals and interpreters would be a useful contribution to the literature and may identify other mechanisms that lead to improved communication for this at-risk group. Further challenges around the deductive nature of the analysis meant we were unable to add weight of meaning to experience per participant, for example the participant either mentioned the experience or not, there was no way of telling whether one participant’s experience was worse or better than another’s.

## Conclusion

Women described interpreter services that are not fit for purpose and do not create a level playing field for pregnant women with social risk factors who do not speak fluent English. Compromised access to interpreter and maternity services across the pregnancy continuum, a lack of choice of interpreter and poor-quality interpretation contributed to inequalities in experiences of maternity care and overall safety. The experiences described provide detailed insight into how poor-quality interpreter services can impact women’s ability to raise concerns, disclose risk factors and communicate effectively with their healthcare providers. The insights provided in the study can inform future practice and research around how maternity services can work to overcome this conflict and may well extend to wider services where women have even less of a voice or access to any interpretation service.

## Supplementary Information

**Additional file 1.** Interview guide with initial programme theories.

## Data Availability

Attached as additional file or contact the lead author HRJ.
