# Supplementary material for: Project20: interpreter services for pregnant women with social risk factors in England: what works, for whom, in what circumstances, and how?
Source: Int J Equity Health. 2021 Oct 24;20:233. doi: 10.1186/s12939-021-01570-8 (PMC8543874; doi:10.1186/s12939-021-01570-8)
Supplement: Supplementary file 1 — Additional file 1. Interview guide with initial programme theories. [file 12939_2021_1570_MOESM1_ESM.docx]

# Supplementary File 1 (S1): - Interview guide with initial programme theories

## Interview guide with initial programme theories

Interviews with women (and family members) 26-28 weeks – 1^st^ interview

| **Question** | **Rationale/ concepts and programme theories addressed** |
| --- | --- |
| **Introduction**  Can you tell me a bit about your pregnancy? How are you feeling? how many weeks pregnant are you? How your pregnancy is going? is this your first pregnancy? | General relaxed introductory questions.  Understanding the general context of women’s lives and how they are experiencing their pregnancy. |
| **Access to maternity services**  When did you first know you were pregnant, and how and when did you get in contact with health services?  How easy was it to get an appointment with a GP/ midwife? What was that appointment like?  Are you able to contact and speak to your GP or midwife easily?  (if relevant) How have you experienced interpreter services? Is it difficult to Have you received written information in your preferred language? | These questions gain context into how women access care and relate to the theoretical framework around Candidacy. Concepts might include: unfamiliarity, help-seeking, resource/barriers, culture.  PT: If maternity care incorporated early pregnancy care (from conception/confirmation of pregnancy), then women would not view it as a package of care for viable and continuing pregnancies and therefore see value of accessing care early in pregnancy to seek support and advice regardless of whether or not they intend to continue the pregnancy  PT: If midwives acknowledged that some women, especially those with unintended pregnancies, are undecided whether they want to continue with the pregnancy when they access services, then they would be better prepared to support and advise women in an objective manner and women would not view maternity services as exclusive to those with continuing pregnancies  PT: If women are able to register with maternity services and GP’s without extensive documentation or evidence of a permanent address, then they could access care earlier in pregnancy, reduce stress and fear of disclosure to agencies or individuals who might put them at risk. This will, in turn, improve early access to abortion services.  PT: If women can access a known midwife 24/7 via a phone call or text message, then they will be better able to engage with services, care will be more personalised, they will feel more cared for, and are less likely to have to repeat their history and experience a variation of responses/advice.  PT: If women receive written information (in their preferred language) about how to access maternity services and what their care will offer and are able to do this directly rather than through a GP, then barriers around NHS administration and/or postal delays will be overcome and antenatal care will commence earlier in pregnancy.  PT: If HCP’s listen to women’s choices about interpreter services, for example a female, an anonymous, or a trusted interpreter, then barriers to their use and effectiveness will be reduced and women would feel more comfortable discussing sensitive subjects and disclosing concerns with their healthcare provider, improving safety.  PT: If women have easy, immediate telephone access to interpreter services to register with services, arrange or reschedule appointments, organise travel to appointments, and access to properly translated materials, then inequity in information received and a key communication barrier will be overcome, and women will be better able to access services. |
| **Perceptions of care**  When did you first meet … (your named midwife)? What was the first meeting like?  Did you choose this type of care? If not, why do you think you were referred to this service? (for example, is it because you live in a certain area/ GP surgery/specific need?)  Did you know you would see the same midwife all the way through your pregnancy? Or did you think your maternity care might be different to this?  Is this the type of care you wanted? How do you feel about it now? What is the best thing about your maternity care? What is not so good? | These questions address access, how women feel about their maternity care, and what type of care they expected to receive. Both context and outcomes may arise from responses.  Concepts might include: discrimination/stigma, unfamiliarity, surveillance, trust, relationships, continuity, HCP characteristics.  Pt: If there are clear paths of communication across different trusts and services such as GP, gynaecology, maternity services, social care and mental health services is seamless, then women would be able to access care earlier in pregnancy and experience less fragmentation and disassociation between the services.  Addressing concepts around choice, trust and surveillance.  PT: If women receive more personal continuity in their care, then they will develop feelings of trust and confidence in their healthcare professionals and have more meaningful interactions (for example disclosing sensitive information or exploring the context of women’s requests/concerns).  PT: If models of care were flexible, appropriately staffed, and midwives had full autonomy over their working days and appointments, then women would not perceive the pressure of time and feel more able to disclose information and midwives would have improved attitudes as they would not be working to unrealistic time constraints.  PT: If women accessing busy maternity services with rushed staff feel that they are being ‘processed through a system by professionals who follow procedures without really noticing the woman in front of them’, then they will not feel cared for, supported, or valued and have a perceived lack of social support. |
| **Previous experiences (if relevant)**  Can you tell me about your other pregnancies? Did you know your midwife/see them all the way through your pregnancy?  Do you think your past experiences of maternity care were good or bad? What made them good/bad?  What about your pregnancies before? What was your birth like? What about after the baby was born?  We know that women who know their midwife/healthcare professional are more likely to have better experiences and outcomes. Has this been the case for you in previous pregnancies? Do you think your maternity care made a difference to these experiences or your pregnancy/birth/early weeks and months with your baby? How did it make a difference? (try to extract the specific mechanisms here) | Rather than test specific programme theories, these questions seek to understand how previous experiences (context) an effect have on how women perceive and experience maternity services. Outcomes will become apparent through discussion of previous experiences of care and clinical pregnancy/birth outcomes etc. The responses will contribute to the discussion around both syndemic and candidacy theory.  Concepts that may arise from these questions include: health inequalities, trust, continuity, relationships, discrimination/stigma.  This question aims to extract specific mechanisms. The question will be asked again at the final interview about this pregnancy, and findings compared. |
| **Social circumstances and support networks**  Can you tell me about your life before your pregnancy? For example, how long have you lived in the country? Who do you live with? How is your general health? Do you see any other healthcare professionals or have a social worker?  (discuss known social risk factors in context, for example if living in poverty discuss the impact of this/what support they have- family/friends/professional/ community)  (If relevant) Do you access other support services? If so, do you find them supportive/useful? Can you tell me about a time where they have or have not been helpful?  Do you trust other support services? (for example, do you have trust in your social worker/ mental health support worker etc)  If you were worried about something, or wanted to talk to somebody who would you go to?  Do you have friends in the local community? Who do you trust in the local community?  Do you feel you can talk to your midwife about personal issues or something that is worrying you?  What is it that makes you feel you can or cannot trust your midwife? | These questions will prompt further insight into the context of women’s lives and add to the discussion around Syndemics. The purpose is to get a sense of the complexity of women’s lives and how this influences their pregnancy, general physical and mental health and experiences of care. Programme theories specific to social risk factor will be highlighted prior to each interview and addressed depending on the woman’s specific social risk factors (See appendix 1 for table of PT’s relating to specific social risk factors).  PT: If women perceive their support network to be invested in their ability to parent successfully, and receive practical, tailored advice and positive affirmations, then they will feel less scrutinised and feel better able to seek support and advice when needed. This in turn will demonstrate how they are able to seek appropriate help and parenting advice.  PT: If women who have had a previous traumatic experience and/or have lost confidence in the system and approach services tentatively are able to develop a trusting relationship with their HCP, then their trust in the system may be restored and their engagement with services improved.  This question aims to reveal the support networks important to women. It addresses the concepts of strengths and assets, trust, relationships, social capital, help-seeking.  PT: If a trusting relationship develops through open discussion and story sharing between women and their HCP, then women will have confidence in their HCP, trust their advice, and benefit from their support.  PT: If women are encouraged by healthcare professionals to raise concerns in an easy and confidential manner and escalate those concerns if they are not satisfied with the response, then they will not only feel empowered and listened to, but potential adverse outcomes could be avoided  PT: If women receive more personal continuity in their care, then they will develop feelings of trust and confidence in their healthcare professionals and have more meaningful interactions (for example disclosing sensitive information or exploring the context of women’s requests/concerns).  PT: If women have the opportunity to get to know their healthcare professional and perceive them to be respectful, understanding, kind, and helpful, then women will feel cared about and cared for, empowered and better able to express or restate their expressed wishes and concerns. Conversely, if women with low socio-economic status experience paternalistic care through being denied choice and perceive HCP’s as lacking warmth, patronising, arrogant, and stigmatising, then they will remain disempowered, feel undervalued and their low self-confidence will increase. |
| What is difficult about your life at the moment?  Do you think this could be improved? How? What would need to happen? What support would be useful?  Do you think your midwife or GP could help with this?  What is the best thing about your life at the moment?  What do you hope for: for this pregnancy? Your baby? life after the baby is born? | These questions press on context in a more direct approach, understanding what is difficult about women’s lives will enable clearer and more specific contexts and outcomes. This will enable exploration of mechanisms in subsequent interviews with women, and the testing of those mechanisms over the course of the pregnancy.  PT: If HCP’s have the time, resources and skills to coordinate and facilitate practical support to meet women’s wider needs (this may include providing information about statutory procedures, contacting social workers, writing letters on their behalf, as well as coordinating, attending and facilitating meetings with other statutory agencies (e.g. Social care, Housing departments, Home Office)), then women will be better informed of unfamiliar processes and better equipped and supported in difficult circumstances.  PT: If HCP’s are educated in maternity benefits available for socially vulnerable women, and able to provide advice around practical matters such as housing, employment, education and care of other children and family members, then women would see more value or purpose in accessing services earlier in pregnancy and further financial hardship and distress for the women could be avoided.  This question revisits the concept of strengths and assets in the woman’s lives and aims to apply a salutogenic approach to the interview process.  This question seeks to identify what outcomes are important to women and will be asked at each interview. |
| Is there anything else you think we should know about how you are experiencing your maternity care so far? | This open probe question enables participants to comment on anything not covered by the interview. The structure of the question keeps the focus on ‘how the programme works’ and ‘in this context’. |

Interviews with women (and family members) 36-38 weeks – 2nd interview

| **­­­** | **Rationale/ concepts and programme theories addressed** |
| --- | --- |
| Can you tell me a bit about how your pregnancy is going? How are you feeling? how many weeks pregnant are you? | General relaxed introductory questions.  Understanding the general context of women’s lives and how they are experiencing their pregnancy. |
| **Engagement with maternity services**  How are you finding your maternity care?  Have you attended all of your appointments?  If not, what was the reason for not attending?  Do you think there are too little/too many appointments?  Was your maternity care scheduled or more relaxed/based on your own needs?  Who decides when and where your appointments are?   - If perceived as a schedule did it meet your needs? Would you prefer to see a midwife as and when you felt you needed to? - Where would you prefer to see your midwife and why?   Do you feel that your maternity care has been flexible? (location/timing)  Do you feel that some appointments have been more important than others?  What is it about appointments that makes you think some are more useful than others? Can you give examples (certain tests? Scans?)  Are you able to get to your appointments easily or would you prefer is a midwife came to you?  -Is cost of public transport expensive  - How long does it take you to get to appts?  -How does this work with childcare commitments?  - Do you feel able to bring your children to appointments?  Would you worry or feel anxious about missing an appointment or having to rearrange an appt?  Have you been able to contact and speak to your GP or midwife easily? What is your preferred method of contacting them and why?  (If relevant) How have you experienced interpreter services? Did you have to ask for them? What is good/not so good about interpreter services?  What is good/not so good about using a family member or friend to interpret for you?  Would you feel happy talking about very personal matters whilst using an interpreter?  Why/why not?  Have you ever used a translator for a telephone conversation? How did you access this?  What do you do if you need to contact a healthcare professional urgently and you do not have anyone around to interpret for you? | If a programme provides physical and social opportunities for women to receive flexible, needs-led care, where the time and place of appointments is co-planned (for example at home, community or a hospital setting), then women will have the best chance to access timely antenatal care, feel listened to and empowered by taking control of their care.  If services are flexible for women who live socially complex lives and move location frequently, or for those who have no access to a telephone or resources to travel far away to a hospital, for example local drop in services, appointments at home, or at the weekend, not at school times for single mothers, not during working hours for women working illegally, then their engagement with services can be improved as much as possible  If midwives are able to visit women at home in the antenatal period, then they will not only overcome barriers such as women unable to travel to appointments, but also be able to assess the living conditions of women to provide more individualised, holistic care.  If antenatal care provides reassurance through clinical checks, effective preparation for labour, an opportunity for socialising with other mothers, and women are encouraged and given the time and resources required to ask questions about their pregnancy and care, then women will see the service as beneficial, feel like active participants and engage with their healthcare providers.  If women have a level of trust and confidence in their HCP’s and do not fear judgement, for example their concerns are listened to on an individual level, they receive meaningful information, and they are able to rebook missed appointments with ease and without reproach, then they will perceive the maternity environment as a place of safety and their engagement with flexible services will improve.  **INTERPRETATION SERVICES**  If HCP’s listen to women’s choices about interpreter services, for example a female, an anonymous, or a trusted interpreter, then barriers to their use and effectiveness will be reduced and women would feel more comfortable discussing sensitive subjects and disclosing concerns with their healthcare provider, improving safety.  If women have easy, immediate telephone access to interpreter services to register with services, arrange or reschedule appointments, organise travel to appointments, and access to properly translated materials, then inequity in information received and a key communication barrier will be overcome, and women will be better able to access services. |
| **Education**  Did you attend any antenatal education class?  If so what was that like? Useful? Culturally sensitive?  Could your partner attend? Did they?  Who gave the class?  What did you learn?  What do you wish you had learnt?  What is given at the right time in your pregnancy?  Did you speak to any other mums there? Do you still speak to anyone now?  How/Where do you access information about pregnancy?  If appropriate: Have you been provided with written information in your language?  If so, by who?  Was it useful?  What could have been better?  If not, would you find this useful? What particular topics would you like it to cover?  During your appointments with your midwife have you learnt things about pregnancy? Birth? Caring for a baby?  Was this useful?  What would you have liked your midwife to teach you/talk to you about?  Have you every changed something that you do because a midwife has taught you about the impact of it? (for example eating healthily? Stopping smoking? Exercise?) Can you give an example? | **EDUCATION**  If antenatal education was culturally sensitive including information that is relevant to women’s individual needs at an appropriate gestation, (for example child friendly settings and classes without the presence of men) and provide an opportunity to meet a small team of midwives providing their care, then more women would engage with the classes and be better informed about their birth choices.  If basic, evidence-based information about maintaining a healthy pregnancy, and procedures/routines is readily available, easy to understand, and translated into new migrant languages, then women would be better informed, able to provide consent, and have less reliance on the internet and advice from friends and family.  If women have more face-to-face time with a health professional to discuss their lifestyle, then they will better understand the impact of risky behaviours, as many do not engage with or understand information provided in leaflets. |
| **Experiences of care**  How has your care been so far?  Have you seen the same midwife throughout your pregnancy?  If so, is that what you wanted form maternity care?  Why?  What is your relationship with your midwife like?  Have you seen the rest of the team? Do you feel like you are familiar with everyone in the team?  Is this the type of care you wanted?  What is the best thing about your maternity care? What is not so good?  Do you think that the team are well staffed? Have they ever seemed over worked or stressed out to you?  Do you trust your midwife? Rest of the team?  Do you feel listened to by your midwife/team?  Do you feel like you are in control of your maternity care? Pregnancy? Are you encouraged to make decisions?  Can you provide an example?  Do you feel like your midwife/team are there to help you or the baby more?  If appropriate: Do you trust social care? Do you think they are a supportive service? Can you tell me any reasons why you think this? Personal experience? Other experiences? Media portrayal?  In your pregnancy so far have you ever told a HCP about something very personal or something that was worrying you?  Would you mind talking about this in more detail?  How did the HCP react? What did they do?/not do?  Is there anything you would have liked them to have do differently?  Can you tell me what characteristics in a midwife/HCP are important to you?  Have you ever had a bad healthcare experience?  Can you tell me about it?  Have you ever experienced discrimination when accessing healthcare?  Can you tell me about it?  Do you feel like your midwife is knowledgeable and sensitive about your culture?  Do you feel like you have a choice in aspects of your maternity care? For example where you give birth?    If you were unhappy with advice given to you or a plan of care from a HCP would you feel able to question it? Would you feel able to decline care/go against advice?  Why/why not? | These questions address how women feel about their maternity care and their relationships with their HCP’s. Both context and outcomes may arise from responses.  Concepts might include: discrimination/stigma, unfamiliarity, surveillance, trust, relationships, continuity, HCP characteristics. Addressing concepts around choice, trust and surveillance.  PT: If women receive more personal continuity in their care, then they will develop feelings of trust and confidence in their healthcare professionals and have more meaningful interactions (for example disclosing sensitive information or exploring the context of women’s requests/concerns).  PT: If models of care were flexible, appropriately staffed, and midwives had full autonomy over their working days and appointments, then women would not perceive the pressure of time and feel more able to disclose information and midwives would have improved attitudes as they would not be working to unrealistic time constraints.  PT: If women who have had a previous traumatic experience and/or have lost confidence in the system and approach services tentatively are able to develop a trusting relationship with their HCP, then their trust in the system may be restored and their engagement with services improved.  PT: If women accessing busy maternity services with rushed staff feel that they are being ‘processed through a system by professionals who follow procedures without really noticing the woman in front of them’, then they will not feel cared for, supported, or valued and have a perceived lack of social support.  If models of care were flexible, appropriately staffed, and midwives had full autonomy over their working days and appointments, then women would not perceive the pressure of time and feel more able to disclose information and midwives would have improved attitudes as they would not be working to unrealistic time constraints.  PT: If women perceive their support network to be invested in their ability to parent successfully, and receive practical, tailored advice and positive affirmations, then they will feel less scrutinised and feel better able to seek support and advice when needed. This in turn will demonstrate how they are able to seek appropriate help and parenting advice  If healthcare professionals give information in an unbiased way, and listen to women’s choices, questions, and decisions, then women will be able to make informed choices about their pregnancy, feel a sense of control and being listened to, and demonstrate their ability to make appropriate choices.  If women feel they are under surveillance, or that asking questions/disclosing information will cause their healthcare provider to judge them, then they will perceive their care to be stressful and disempowering, rather than a supportive, informative preparation to parenthood and will feel that it is safer not to ask for help.  PT: If women receive more personal continuity in their care, then they will develop feelings of trust and confidence in their healthcare professionals and have more meaningful interactions (for example disclosing sensitive information or exploring the context of women’s requests/concerns).  If women are offered continuity of care and are able to build a trusting relationship with their midwife, then underlying social risk factors can be explored and care individualised to their needs to improve engagement and empowerment so that women are better able to express or restate their expressed wishes and concerns.  PT: If women have the opportunity to get to know their healthcare professional and perceive them to be respectful, understanding, kind, and helpful, then women will feel cared about and cared for, empowered and better able to express or restate their expressed wishes and concerns.  Conversely, if women with low socio-economic status experience paternalistic care through being denied choice and perceive HCP’s as lacking warmth, patronising, arrogant, and stigmatising, then they will remain disempowered, feel undervalued and their low self-confidence will increase.  If women with low socioeconomic status experience discriminatory, or impersonal care, then their often already fragile self-confidence can be further undermined, making them feel they are not good enough to parent.  If midwives acknowledge the importance of culture and the influence of family members on women’s experience of pregnancy, then they will be able to personalise care around the needs and cultural norms of the family unit and avoid potential conflicts in offering advice that does not reflect a cultural norm  If HCP’s work within a community where they are immersed in local cultures different to their own, or the hospital environment, then they will become culturally sensitive, women will not feel their cultural needs are being disregarded in favour of the western medical model and inequities in access, engagement, the uptake of screening, and antenatal education will be reduced.  If healthcare professionals inform women of their right to choice, through education and providing the evidence-based information women need to exercise that choice, then they will be empowered and their self-confidence increased through shared decision making, and would not feel as through accessing care equates to relinquishing control through perceptions of manipulation and coercion by the healthcare professional.  If maternity care encompasses the foundations of woman-centred care: working with women as partners, respecting their expertise of their own body, needs and baby, and making decisions based upon individuals rather than stereotypes or entrenched professional norms, then women will be more situated in a context of control rather than disempowerment. For some women this may also avoid disempowerment, feelings of being pressurised, ignored and excluded, long lasting psychological trauma, and increase bonding between a mother and her baby.  If healthcare professionals recognise that socially deprived women are more likely to experience paternalistic maternity care, as passive recipients, then the HCP can personalise care and strive to involve women in planning and decision making to ensure women are active, respected participants. This can in turn improve the self-confidence these women often lack in situations where there is a power imbalance.  If women are encouraged by healthcare professionals to raise concerns in an easy and confidential manner and escalate those concerns if they are not satisfied with the response, then they will not only feel empowered and listened to, but potential adverse outcomes could be avoided. |
| **Social circumstances/ practical support**  The last time we met we spoke about some of the difficulties you were having with……  How is this situation now?  Has your midwife been able to help with this in any way? Please give examples  Do you think your midwife has a lot of knowledge about (housing issues/benefits available/ local community support)  What about other HCP’s or support services?  Do you think this has had an effect on your levels of stress?  Do you think this has had an effect on your pregnancy? How? Please give examples  Is there anything you were worried about being able to afford that your midwife has helped you get? Or told where to go to find more affordable things? Can you give an example?  Is there anything you still worry that you do not have for the baby? | Rather than test specific programme theories, these questions seek to understand how women’s current social circumstances have an effect have on how women perceive and experience maternity services. The responses will contribute to the discussion around both syndemic and candidacy theory. These questions aim to extract specific mechanisms and get a sense of the complexity of women’s lives and how this influences their pregnancy, general physical and mental health and experiences of care.  PT: If women feel they have a continued supportive presence throughout pregnancy and the perinatal period, either with a midwife, GP or other healthcare professional, then they will feel better supported and have reduced feelings of anxiety, increased sense of control, and enhanced self-beliefs and wellbeing.  If HCP’s work in a small geographical area where they are visible and become known by other members of the community, religious networks and other ‘gatekeepers’, then they can work together to develop trust, facilitate family and community-centred care, and educate the community with evidence-based information and dispel common, harmful myths.  If HCP’s are familiar with local charities, food banks, befriending programmes and support services then they will be able to introduce women to these services in order to provide the most supportive networks possible before they are discharged from maternity care and women will be better able to integrate into the community.  If women perceive their support network to be invested in their ability to parent successfully, and receive practical, tailored advice and positive affirmations, then they will feel less scrutinised and feel better able to seek support and advice when needed. This in turn will demonstrate how they are able to seek appropriate help and parenting advice.  PT: If HCP’s have the time, resources and skills to coordinate and facilitate practical support to meet women’s wider needs (this may include providing information about statutory procedures, contacting social workers, writing letters on their behalf, as well as coordinating, attending and facilitating meetings with other statutory agencies (e.g. Social care, Housing departments, Home Office)), then women will be better informed of unfamiliar processes and better equipped and supported in difficult circumstances.  PT: If HCP’s are educated in maternity benefits available for socially vulnerable women, and able to provide advice around practical matters such as housing, employment, education and care of other children and family members, then women would see more value or purpose in accessing services earlier in pregnancy and further financial hardship and distress for the women could be avoided.  If midwives and women are able to get to know each other and build a trusting relationship, then the midwife will be more aware of a woman’s social situation and able to provide individualised, holistic support without labelling women or making assumptions about their needs based on a perceived cultural background. |
| Social Risk Factor questions (see last page) | Programme theories specific to social risk factor will be highlighted prior to each interview and addressed depending on the woman’s specific social risk factors (See Appendix 1 for table of PT’s relating to specific social risk factors). This question aims to reveal the support networks important to women. It addresses the concepts of strengths and assets, trust, relationships, social capital, help-seeking. |
| **MULTIDISCIPLINARY WORKING/COMMUNICATION**  **Do you see other HCP’s or access support services?**  **What are they?**  **Do you think that your midwife or midwifery team communicates with these services?**  **Do they do this well?**  **Is this something you think is important?**  **Does your midwife attend other appointments with HCP’s? for example obstetric appts or Social care meetings?**  **Can you give an example?**  **Do you think this is something that is important?** | If there are clear paths of communication across different trusts and services such as GP, gynaecology, maternity services, social care and mental health services is seamless, then women would be able to access care earlier in pregnancy and experience less fragmentation and disassociation between the services.  If models of care facilitated the development of effective support networks for women throughout their pregnancy through working with family members and multidisciplinary support services (social workers, health visitors, support workers, children’s centres and voluntary sector agencies), then that established support network will enable new mothers to flourish and become confident and successful parents.  If a programme offers advocacy, midwife attendance at meetings, and other forms of emotional support during interactions with social care then women will feel supported and informed of unfamiliar processes |
| Is there anything else you think we should know about how you are experiencing your maternity care so far? | This open probe question enables participants to comment on anything not covered by the interview. The structure of the question keeps the focus on ‘how the programme works’ and ‘in this context’. |

Interviews with women (and family members) Postbirth/ 6 weeks postnatal – 3rd interview

| **Question** | **Justification/ Programme theory** |
| --- | --- |
| How are you? How old is baby now?  How are things currently going for you? |  |
| (If relevant) Interpreter services – Emergency care/Labour and postnatal  Did you have access to interpreter services in labour/after your baby was born? Did you have to ask for them? If no how did you find this service? If not, why not?  What is good/not so good about using interpreter services/ family member/ friend to interpret for you?  Would you feel happy talking about very personal matters whilst using an interpreter?  Why/why not?  Have you ever used a translator for a telephone conversation. How did you access this?  What do you do if you need to contact a healthcare professional urgently (for example in early labour) and you do not have anyone around to interpret for you? | If women have easy, immediate telephone access to interpreter services to register with services, arrange or reschedule appointments, organise travel to appointments, and access to properly translated materials, then inequity in information received and a key communication barrier will be overcome, and women will be better able to access services. |
| Practical support  Have you received helpful, practical support during pregnancy and after your babies birth? (give examples)  How did that make you feel?  Has this helped you overcome difficulties you were having? | If HCP’s support women in difficult circumstances to address the emotional and practical challenges they face by providing them with new skills, knowledge and resources (for example help to resolve infant feeding challenges, provision of breast pumps, bottles and storage bags, reassurance, and motivation to abstain from illegal substances), then they will be better prepared to overcome challenges and internalise this as evidence of care and concern that HCP’s feel towards them. |
| Continuity  Have you seen the same midwife throughout pregnancy? Did they care for you during labour? Did you know the person caring for you during labour?  Birth: Who did you contact? How was the birth experience?  Did you feel well prepared for labour and birth? Why do you think this was?  Who provided your postnatal care?  How many times were you seen by a midwife after baby was born?  What was your care on the postnatal ward like? Were you visited by your midwife? Who discharged you?  Who did the baby check? | If women can access a known midwife 24/7 via a phone call or text message, then they will be better able to engage with services, care will be more personalised, they will feel more cared for, and are less likely to have to repeat their history and experience a variation of responses/advice.  If women feel they have a continued supportive presence throughout pregnancy and the perinatal period, either with a midwife, GP or other healthcare professional, then they will feel better supported and have reduced feelings of anxiety, increased sense of control, and enhanced self-beliefs and wellbeing.  If women are offered continuity of care and are able to build a trusting relationship with their midwife, then underlying social risk factors can be explored and care individualised to their needs to improve engagement and empowerment so that women are better able to express or restate their expressed wishes and concerns. |
| Community/Location  Where did you have most of your appointments during pregnancy?  Is this what you wanted?  Where would you prefer to have your appointments?  Have you used your local children’s centre at all? Who introduced you to this?  What about other local resources/charities/support groups?  Do you feel like you have a supportive network of people around you? If so, who does this consist of? | If HCP’s are familiar with local charities, food banks, befriending programmes and support services then they will be able to introduce women to these services in order to provide the most supportive networks possible before they are discharged from maternity care and women will be better able to integrate into the community.  If a programme provides physical and social opportunities for women to receive flexible, needs-led care, where the time and place of appointments is co-planned (for example at home, community or a hospital setting), then women will have the best chance to access timely antenatal care, feel listened to and empowered by taking control of their care. |
| **Communication/Choice/Agency**  During your pregnancy/birth and postnatal care do you feel you were given the time and opportunity to ask questions?  Do you have any examples of this?  Do you feel like you were well informed about pregnancy/birth etc? Were you provided with appropriate information?  Did you feel involved in decisions about your pregnancy care? (place of birth, pain relief, baby care)  Do you think that your knowledge about your own body/needs/baby is respected by heathcare professionals?  Would you feel able to question care that you were concerned about/not happy about?  If not, why?  Have you ever felt pressurised into doing something you didn’t want to do?  Have you ever felt your needs/wishes/choices were ignored by healthcare professionals?  Do you have any examples of this happening? | If antenatal care provides reassurance through clinical checks, effective preparation for labour, an opportunity for socialising with other mothers, and women are encouraged and given the time and resources required to ask questions about their pregnancy and care, then women will see the service as beneficial, feel like active participants and engage with their healthcare providers.  If models of care facilitated the development of effective support networks for women throughout their pregnancy through working with family members and multidisciplinary support services (social workers, health visitors, support workers, children’s centres and voluntary sector agencies), then that established support network will enable new mothers to flourish and become confident and successful parents.  If healthcare professionals recognise that socially deprived women are more likely to experience paternalistic maternity care, as passive recipients, then the HCP can personalise care and strive to involve women in planning and decision making to ensure women are active, respected participants. This can in turn improve the self-confidence these women often lack in situations where there is a power imbalance.  If women are encouraged by healthcare professionals to raise concerns in an easy and confidential manner and escalate those concerns if they are not satisfied with the response, then they will not only feel empowered and listened to, but potential adverse outcomes could be avoided.  If healthcare professionals inform women of their right to choice, through education and providing the evidence-based information women need to exercise that choice, then they will be empowered and their self-confidence increased through shared decision making, and would not feel as through accessing care equates to relinquishing control through perceptions of manipulation and coercion by the healthcare professional.  If maternity care encompasses the foundations of woman-centred care: working with women as partners, respecting their expertise of their own body, needs and baby, and making decisions based upon individuals rather than stereotypes or entrenched professional norms, then women will be more situated in a context of control rather than disempowerment. For some women this may also avoid disempowerment, feelings of being pressurised, ignored and excluded, long lasting psychological trauma, and increase bonding between a mother and her baby. |
| Surveillance/ Trust  Did you have any involvement with social care/ do you have a social worker?  If so, do you know the reason for this?  Have they been helpful/supportive?  How did you feel about being referred to social care?  How do you feel about it now?  Do you trust your social worker?  Would you tell your social worker about something that was worrying you?  If so do you have any examples of this?  Has your midwife been involved in your social care? If so how?  How did you feel about this? | If women feel they are under surveillance, or that asking questions/disclosing information will cause their healthcare provider to judge them, then they will perceive their care to be stressful and disempowering, rather than a supportive, informative preparation to parenthood and will feel that it is safer not to ask for help.  If women perceive their support network to be invested in their ability to parent successfully, and receive practical, tailored advice and positive affirmations, then they will feel less scrutinised and feel better able to seek support and advice when needed. This in turn will demonstrate how they are able to seek appropriate help and parenting advice.  If women receive more personal continuity in their care, then they will develop feelings of trust and confidence in their healthcare professionals and have more meaningful interactions (for example disclosing sensitive information or exploring the context of women’s requests/concerns). |
| Culture  Has your midwife asked you about your culture? Do you think she is knowledgeable about your culture?  Do you have any examples of this?  Do you feel your midwife is knowledgeable about your local community (for example support services available?)  Do you feel you have ever been treated differently because of your race/age/class etc?  Do you have any more information you think would be useful for us to know about your maternity care experience? (give examples)  Thank you | If midwives acknowledge the importance of culture and the influence of family members on women’s experience of pregnancy, then they will be able to personalise care around the needs and cultural norms of the family unit and avoid potential conflicts in offering advice that does not reflect a cultural norm  If HCP’s work within a community where they are immersed in local cultures different to their own, or the hospital environment, then they will become culturally sensitive, women will not feel their cultural needs are being disregarded in favour of the western medical model and inequities in access, engagement, the uptake of screening, and antenatal education will be reduced.  If women with low socioeconomic status experience discriminatory, or impersonal care, then their often already fragile self-confidence can be further undermined, making them feel they are not good enough to parent. |
